# Ten-Year Results After Canaloplasty and Phacocanaloplasty

**DOI:** 10.3390/jcm14072481

**Published:** 2025-04-04

**Authors:** Hanga Beres, Bendegúz Gyarmati, Simona Gurzu, Gabor Bernd Scharioth

**Affiliations:** 1 Aurelios Augenzentrum, Erlbruch, 34–36, 45657 Recklinghausen, North Rhine-Westphalia, Germany; bendeguz.gyarmati@augenzentrum.org (B.G.); gabor.scharioth@augenzentrum.org (G.B.S.); 2 Doctoral School of Medicine and Pharmacy, George Emil Palade University of Medicine Pharmacy Science and Technology of Targu Mures, Gheorghe Marinescu 38, 540139 Targu Mures, Romania; simonagurzu@yahoo.com; 3 Department of Pathology, George Emil Palade University of Medicine Pharmacy Science and Technology of Targu Mures, Gheorghe Marinescu 38, 540139 Targu Mures, Romania; 4 Department of Ophthalmology, University of Szeged, Dugonics Square, 13, H-6720 Szeged, Hungary

**Keywords:** glaucoma, glaucoma surgery, canaloplasty, iTrack, glaucolight, long-term follow-up, intraocular pressure

## Abstract

**Background/Objectives**: To evaluate the long-term efficacy and safety of canaloplasty and phacocanaloplasty in patients with primary open-angle glaucoma (POAG) and pseudoexfoliation glaucoma (PEXG). **Methods**: This retrospective observational study included 85 patients with POAG and PEXG who underwent canaloplasty (group 1) or phacocanaloplasty (group 2). Every patient had complete medical records over a 10-year follow-up period. The primary endpoints were the pressure-lowering and drug-sparing effects. The secondary endpoints were intra- and postoperative complications as well as the need for additional surgical interventions. **Results**: In group 1, the mean baseline intraocular pressure (IOP) of 22.1 ± 0.9 mmHg was reduced to 15.3 ± 0.5 mmHg, 15.7 ± 0.5 mmHg, and 15.9 ± 0.7 mmHg at 1, 5, and 10 years, respectively. The mean medication use decreased from 2.4 ± 1.0 before surgery to 0.1 ± 0.5, 0.8 ± 1.1, and 1.4 ± 1.3 at 1,5, and 10 years, respectively. In group 2, IOP was reduced from 20.4 ± 1.5 to 15.6 ± 1.0, 14.3 ± 0.8, and 14.2 ± 1.2 at 1, 5, and 10 years, respectively. The mean medication use dropped from 2.4 ± 1 to 0.3 ± 0.9, 0.9 ± 1.4, and 0.8 ± 1.1 at 1,5, and 10 years, respectively. Goniopuncture was performed postoperatively in nine cases (13.9%) within the initial 3 months due to IOP spikes (POAG *n* = 6, PEXG *n* = 3). Patients with PEXG had a significantly higher likelihood of requiring re-operation (HR = 5.11, HR = 5.11, 95% CI 1.05–24.74, *p* = 0.043). No serious complications were observed. **Conclusions**: Canaloplasty is a safe and effective procedure for lowering IOP in eyes with POAG and PEXG, achieving approximately a 30% reduction in IOP. PEXG patients are likelier to have IOP spikes in the late postoperative period therefore careful monitoring and management is required.

## 1. Introduction

Open-angle glaucoma (OAG) represents a considerable challenge in ophthalmology, characterized by the insidious onset of chronic and irreversible optic neuropathy. Elevated intraocular pressure (IOP) in glaucoma is caused by an increase in aqueous humor resistance on its drainage pathways [1], mainly at the level of the juxtacanalicular trabecular meshwork (TM) and inner wall of Schlemm’s canal (SC) [1,2]. Surgical interventions Fthat target the conventional pathway, aiming to restore the trabeculocanalicular outflow, have been gaining more interest in the past decades [3]. Canaloplasty is a nonpenetrating surgical technique that targets outflow resistance at the level of SC and collector channels, having an additional effect at the inner wall of the SC and the TM [4]. Since its first introduction in the year 2006, many previous studies have found that canaloplasty has a good safety profile and effectively lowers IOP in patients with OAG [5,6,7]. It is generally indicated for patients diagnosed with early to medium-advanced OAG where a pressure of 13–15 mmHg is targeted [8].

Patients with longstanding glaucoma frequently face a reduction in visual acuity caused by concomitant cataract formation. As performing a combined surgery of canaloplasty with phacoemulsification does not seem to impact negatively postoperative IOP outcomes as in the case of the trabeculectomy [9], it is generally up to the surgeon to decide if a combined or a two-step procedure is better suited for the patient. According to some related studies in the literature, canaloplasty and phacocanaloplasty can provide a comparable IOP reduction [10,11,12], with phacocanaloplasty being somewhat superior for target IOPs ≤ 21 and ≤18 but not for target IOPs lower than 16 mmHg [12]. However, after phacocanaloplasty, IOP peaks were seen more frequently in the first postoperative weeks. [12]. Pseudoexfoliation glaucoma and cataract can present greater surgical challenges both intraoperatively and postoperatively. For this reason, some authors recommend alternative IOP-lowering procedures [13].

Despite the promising results of both canaloplasty and phacocanaloplasty, several gaps remain in the literature. First, most studies focused on short- to mid-term outcomes with limited data on long-term efficacy and the potential need for repeat surgeries. There is also a need for a more robust analysis of the factors that influence the success of canaloplasty, such as the specific surgical techniques employed, patient selection criteria, and glaucoma subtypes. This study aims to contribute to the growing body of literature on canaloplasty by addressing these gaps, particularly focusing on long-term outcomes and the comparative effectiveness of canaloplasty versus phacocanaloplasty in patients with primary open-angle glaucoma (POAG) or pseudoexfoliation glaucoma (PEXG).

## 2. Materials and Methods

### 2.1. Design

Our research presents the decade-long findings of a single-center retrospective chart review assessing the outcomes of canaloplasty at Aurelios Augenzentrum, Recklinghausen, Germany. The procedures were carried out by a single surgeon (G.B.S.) between January 2008 and December 2013. The study cohort comprised 88 eyes diagnosed with POAG and PEXG. All research was conducted in compliance with the principles outlined in the Declaration of Helsinki. The protocol was approved by the Ethics Committee of Westfalen-Lippe, Germany.

All patients provided signed informed consent. Presurgery data of the last eye examination, before surgery, included Goldmann applanation tonometry, medication use, slit lamp biomicroscopy, and funduscopy. Postoperative follow-up examinations were at 1 day, 1 week, and 1, 3, 6, 9, and 12 months. After the first year, patients came to 3–6 monthly exams, depending on their IOP status. At each follow-up visit, all pertinent data were documented, including IOP measurements, slit-lamp examination findings, gonioscopy results, prescribed ophthalmic medications, and any adverse events. The primary endpoints were the average IOP and the mean number of glaucoma medications recorded at each follow-up. Secondary endpoints encompassed surgical and post-surgical complications and the need for secondary reinterventions such as laser goniopuncture or surgical reintervention.

### 2.2. Patient Selection

All participants were at least 18 years old at the time of enrollment and capable of giving informed consent, and were scheduled for glaucoma surgery or combined cataract and glaucoma surgery. Inclusion criteria required a diagnosis of POAG or PEXG. Patients with advanced glaucoma and visual field loss were not excluded, nor were those with a baseline IOP of less than 16 mmHg. The protocol permitted prior surgeries provided they did not interfere with the complete circumferential catheterization of SC. To ensure consistency, only patients who completed all follow-up visits at our clinical practice for the entire follow-up period were included. Patients who were lost to follow-up or deceased were excluded from this study.

### 2.3. Surgical Technique

Canaloplasty was carried out utilizing either an iTrack™ microcatheter (iTrack 250, Ellex iScience, Inc., Freemont, CA, USA) or a Glaucolight microcatheter (D.O.R.C. Dutch Ophthalmic Research Center, Zuidland, The Netherlands) to circumferentially probe the SC. The procedure began by creating a limbal conjunctival opening, followed by the dissection of a superficial scleral flap approximately 5 × 5 mm in size. A paracentesis was then performed, and a deep scleral flap measuring approximately 4.5 × 4 mm was carefully dissected just above the choroid. The trabeculo-descemetic window measuring 1–1.5 mm was prepared, and the SC was dissected. Subsequently, the deep scleral flap was excised. The ostia of the SC were viscodilated using a high-viscosity ophthalmic viscoelastic device (OVD, Healon GV; Johnson and Johnson, New Brunswick, NJ, USA) delivered through a microcannula. Circumferential probing of the canal was then performed using the iTrack or Glaucolight microcatheter. Once the tip of the microcatheter emerged, it was tied with a 10/0 polypropylene suture and withdrawn, threading the suture into the canal. The suture was subsequently secured with a four-throw knot under tension. Before closing the superficial flap, high-viscosity OVD was injected into the ostia, and additional OVD was placed under the scleral flap upon closure. The conjunctiva was closed water-tight, and the anterior chamber (AC) was filled with balanced salt solution to restore normal IOP [14].

**Figure 1:**
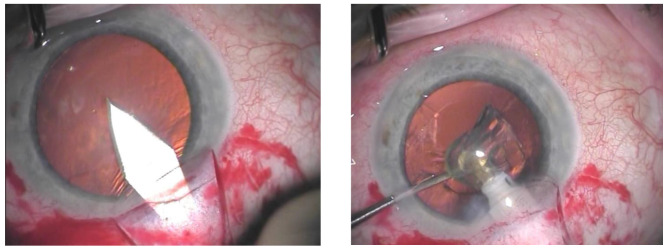
Main incision through the shared incision (on the **left**); phacoemulsification carried out through the shared incision (on the **right**).

When phacocanaloplasty was performed, phacoemulsification was carried out through a shared incision (Figure 1). After preparing the superficial scleral flap, the deep scleral flap was dissected to expose SC. The phaco incision was positioned between the deep and superficial flaps, and a standard microincision cataract surgery (MICS) procedure was performed. Following intraocular lens (IOL) implantation, the OVD was retained in the AC, after which canaloplasty was carried out. Finally, the OVD was removed from the AC after the closure of the superficial flap using bimanual irrigation-aspiration [14].

### 2.4. Statistical Analysis

The primary endpoints in this study included the IOP and the number of antiglaucoma medications at various time points: 1, 3, 6, 12 months, as well as annually up to 10 years postoperatively. The secondary endpoints included surgical/postsurgical complications as well as secondary interventions. Descriptive statistics (mean, standard deviation, count, percentage) were used to summarize the demographic and clinical characteristics of the study cohort. To assess the association between qualitative variables, the Chi-squared test and Fisher test were used. Postoperative changes in IOP were assessed using (generalized) linear mixed models (LMM). Model assumptions were validated by assessing residual normality using Q-Q plots and ensuring no significant multicollinearity (variance inflation factor < 5). To compare IOP across surgical groups (phakic canaloplasty, phakic phacocanaloplasty, and pseudophakic canaloplasty), post hoc pairwise comparisons were conducted using the Bonferroni test. To evaluate postoperative medication change, Poisson generalized LMM was used. To account for zero counts, a pseudo-count of 1 was added to all compound count values. Patients who required reoperations, subsequent data on IOP, and other variables were excluded from the analyses. To further evaluate risk factors that may influence reoperation risk (e.g., glaucoma type, preoperative IOP, and medications), Cox proportional hazards regression was used. Kaplan–Meier survival analysis was used to evaluate the time to event in both surgical cohorts. The significance threshold was set at a *p*-value of 0.05. The statistical analysis was performed with SPSS Statistics 26.0.0.

## 3. Results

### 3.1. Clinicopathological Data

Table 1 summarizes the baseline data. The study cohort consisted of 88 patients diagnosed with primary open-angle glaucoma (*n* = 77) and pseudoexfoliation glaucoma (*n =* 8). The cannulation success rate was 96.6%; patients with failed cannulation (*n* = 3) were excluded from the statistical analysis.

The mean age of the cohort was 68.8 ± 8.1 years. All patients were white, and the majority were female. Most of the patients (90.6%) were diagnosed with POAG.

### 3.2. Canulation

Canaloplasty was performed in 37 cases using the iTrack microcatheter (iTrack 250, Ellex iScience, Inc., Freemont, CA, USA) (Canaloplasty *n* = 27, Phacocanaloplasty *n* = 10) and in 48 cases using the Glaucolight (D.O.R.C. Dutch Ophthalmic Research Center, Zuidland, The Netherlands) (Canaloplasty *n* = 38, Phacocanaloplasty *n* = 10). The LMM showed that the choice of microcatheter (iTrack vs. Glaucolight) significantly affects IOP overall (*p* = 0.004), and this effect varies by procedure type (CP vs. CP+Phaco, *p* = 0.001). Glaucolight reached a slightly lower IOP in the CP group (mean difference 0.347 mmHg) and a significantly lower IOP in the CP+Phaco group (mean difference 3.536 mmHg). The IOP trajectories over time do not differ by catheter type (*p* = 0.726) or vary by procedure type over time (*p* = 0.269). Intraoperative complications were more frequent in the iTrack group, with higher incidences of Descemet membrane perforation (*n =* 6 vs. *n* = 2), Descemet membrane detachment (*n =* 3 vs. *n =* 0), but no anterior chamber perforation (*n* = 0 vs. *n* = 1).

### 3.3. Change in Intraocular Pressure and Antiglaucoma Medication

**Figure 2:**
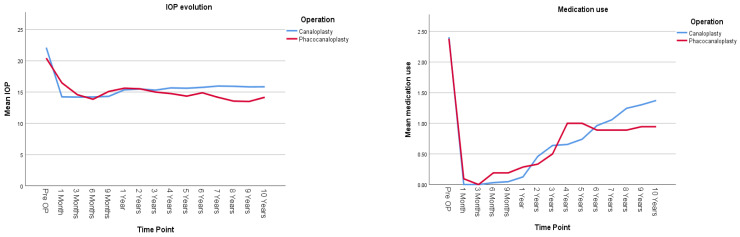
Illustrates postoperative IOP evolution (on the **left**) and medication use (on the **right**) in all studied eyes undergoing either canaloplasty (CP) or phacocanaloplasty (CP+Phaco). Values represent adjusted means from a linear mixed model, adjusted for time, lens status, and exclusion of reoperation cases.

Figure 2 and Table 2 present the IOP evolution and the number of hypotensive medications used for the canaloplasty (CP) and phacocanaloplasty (CP+Phaco) groups during the 10-year follow-up period. There was no statistically significant difference between the IOP-lowering effect of CP and CP+Phaco; both operation types show a similar pattern of IOP change, and the overall average IOP does not differ significantly between them (mean difference = 0.666 mmHg, *p* = 0.207). Lens status does not significantly impact IOP in the canaloplasty group, either in terms of overall IOP levels (*p* = 0.535) or how IOP changes over time (*p* = 0.249), pseudophakic patients had on average 0.363 mmHg higher IOP levels (95% CI [−0.793, 1.519]) than phakic ones. Both phakic and pseudophakic patients in the CP group show similar IOP trends, with a significant reduction over time (*p* = 0.000).

Medication use significantly decreased over time following both CP and CP+Phaco (*p* < 0.001). However, the type of surgery did not have a significant overall effect on medication dependence (*p* = 0.926).

**Figure 3:**
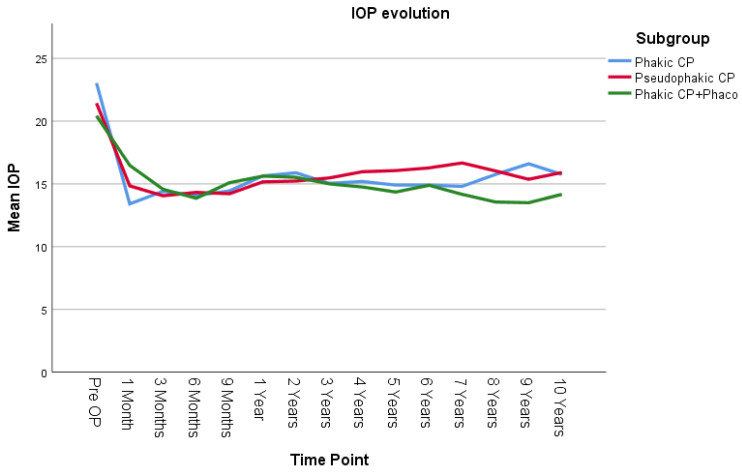
IOP evolution in the 3 surgical subgroups. Phakic CP: phakic patients after canaloplasty, Pseudopakic CP: pseudophakic patients after canaloplasty, Phakic CP+Phaco: phakic patients after phacocanaloplasty. Values represent adjusted means from a linear mixed model, adjusted for time, lens status, and exclusion of reoperation cases.

IOP was compared using the LMM across three groups: phakic patients undergoing canaloplasty (Phakic CP), pseudophakic patients undergoing canaloplasty (Pseudophakic CP), and phakic patients undergoing combined canaloplasty and phacoemulsification (Phakic CP+Phaco) (Figure 3). There was no statistically significant difference in the overall average IOP among the three groups across all time points (*p* = 0.451). There was also no statistically significant difference in how IOP changes over time among the three groups (*p* = 0.311). Pairwise comparisons across the three groups also showed no significant differences. The largest observed mean IOP difference was 0.712 mmHg (95% CI [−0.89, 3.24]) between the Pseudophakic CP and Phakic CP+Phaco group, but this difference was not statistically significant (*p* = 0.657, Bonferroni-adjusted).

### 3.4. Surgical Success

Complete success was defined as achieving an IOP of 18 mm Hg or lower without the use of glaucoma medications at a given postoperative time point. Qualified success was defined as an IOP of 18 mm Hg or lower with the use of medications. Failure was defined as the time until additional glaucoma surgery was performed or having an IOP over 18 mmHg at two consecutive follow-up visits (at least 3 months after surgery).

Table 3 shows the success results for both groups. The complete success rate of group 1 was 89.2%, 49.2%, and 29.2% after 1, 5, and 10 years, respectively. Qualified success in group 1 was achieved in 95.4%, 80.0%, and 66.2% after 1, 5, and 10 years, respectively. The complete success rate of group 2 was 85%, 50%, and 45% after 1, 5, and 10 years, respectively. The qualified success rate of group 2 was 90%, 75%, and 70% after 1, 5, and 10 years, respectively.

**Figure 4:**
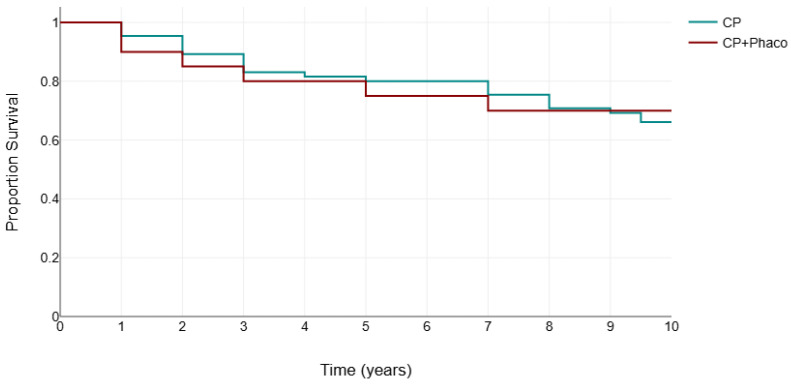
Kaplan–Meier survival analysis for canaloplasty (CP) and phacocanaloplasty (CP+Phaco).

Figure 4 presents the Kaplan–Meier survival analysis for both groups, using failure criteria defined as either the need for additional glaucoma surgery or two consecutive follow-up visits with an IOP exceeding 18 mm Hg, with or without glaucoma medication use.

### 3.5. Complications and Reinterventions

In nine cases, intraoperative complications were seen. They included Descemet perforation (*n* = 8), with or without iris prolapse, Descemet membrane detachment (DMD, *n* = 3), and perforation in the anterior chamber (*n* = 1) recorded. In cases with iris prolapse, an iridectomy was performed. As for the postoperative complications, the patients with DMD developed a hemorrhagic Descemet membrane detachment (HDMD) inferiorly. As none of them involved the visual axis observation was chosen as the primary management approach. Additionally, two patients developed a filtration bleb postoperatively. No other serious postoperative complications were recorded.

**Figure 5:**
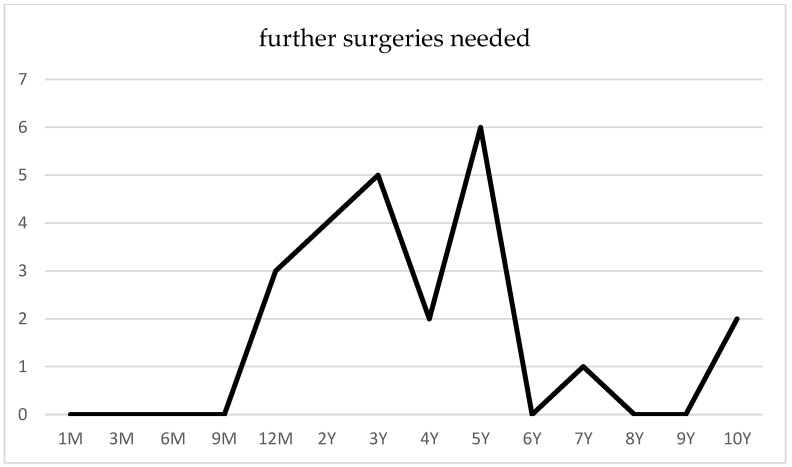
Surgical reinterventions following canaloplasty.

Following canaloplasty, goniopuncture was performed postoperatively in nine cases (13.9%) within the initial 3 months due to IOP spikes (POAG *n* = 6, PEXG *n* = 3). Conversely, no instances of goniopuncture were documented after phacocanaloplasty. A total of 16 eyes required at least one reoperation, comprising 12 POAG cases and 4 PEXG cases, predominantly within the initial 5 years following canaloplasty (Figure 5). Secondary intervention was indicated for patients with an IOP exceeding 21 mmHg despite maximal tolerated topical therapy and by the presence of morphological changes. These patients underwent one of the following procedures: ab interno trabeculotomy, Baerveldt shunt implantation, or cyclophotocoagulation.

To evaluate the factors influencing the likelihood of reoperation, a Cox proportional hazards regression analysis was performed. The only statistically significant covariate was the glaucoma subtype, PEXG, which significantly increased the risk of reoperation (*p* = 0.043, HR = 5.11, CI 1.05–24.74). Patients with PEXG are much more likely to require reoperation compared to those with POAG. However, this finding may be limited by the small sample size of PEXG patients (*n* = 8), potentially impacting statistical power.

Phacocanaloplasty was associated with a 40% lower hazard of reoperation compared to canaloplasty alone; however, this result was not statistically significant (*p* = 0.489). Pseudophakic patients had a 20% higher hazard of reoperation compared to phakic patients, which was also not statistically significant (*p* = 0.784). Each additional preoperative medication increased the hazard of reoperation by 53%, though this result did not reach statistical significance (*p* = 0.156). Intraoperative complications were associated with a 41% reduced hazard of reoperation, but this effect was not statistically significant (*p* = 0.565). Each additional year of age increased the hazard of reoperation by 4%, also not statistically significant (*p* = 0.335). Female patients had a 40% lower hazard of reoperation compared to male patients, but this finding was not statistically significant (*p* = 0.373). Each 1 mmHg increase in preoperative IOP was associated with a 7% higher hazard of reoperation. However, this finding was not statistically significant (*p* = 0.123).

## 4. Discussion

In this single-center retrospective study, ab externo canaloplasty, performed as a standalone procedure or with cataract surgery, proved to be a safe and effective option for long-term IOP reduction in POAG and PEXG patients. Over 10 years, IOP remained approximately 30% lower than preoperative levels, while medication dependency significantly decreased but showed a gradual increase over time.

Recently, Ennerst et al. published a study comparing canaloplasty (Group A, *n* = 28) and phacocanaloplasty (Group B, *n* = 20) over an 11-year period [15]. Similarly to our findings, their study found no significant difference in the IOP-lowering effectiveness of canaloplasty and phacocanaloplasty. When comparing surgical success rates, Ennerst et al. reported higher long-term complete success rates (IOP ≤ 18 mmHg without medication), with 41.7% for canaloplasty and 66.7% for phacocanaloplasty at 10 years, while our study found 29.2% and 45%, respectively. Qualified success rates (IOP ≤ 18 mmHg with or without medication) were also higher in their study (83.3% for canaloplasty and 91.7% for phacocanaloplasty at 10 years) compared to our findings (66.2% and 70%, respectively). Additionally, Ennerst et al. reported greater medication independence, with reductions from 1.5 ± 1 to 0.9 ± 1.1 in Group A and from 2.2 ± 1.2 to 0.5 ± 0.9 in Group B at 10 years. In comparison, our cohort exhibited a higher baseline medication dependency (2.4 vs. 1.5 for CP; 2.4 vs. 2.2 for CP+Phaco), which may have contributed to the lower long-term success rates. They also reported lower reoperation rates (10% vs. 18.8% in our study), possibly due to a more homogeneous cohort excluding PEXG patients, who are at higher risk for IOP decompensation as observed in our study. Differences in baseline characteristics such as preoperative IOP, medication use, patient selection, and sample sizes may explain the variations in percentage reductions and absolute outcomes between the two studies.

Several prospective and retrospective studies with mid-term follow-ups have reported mean IOP reductions ranging from 28% to 35%, with IOP values between 15 and 15.5 mmHg and medication use ranging from 0.5 to 0.9 for canaloplasty [6,7,11,16]. Our study results are consistent with these findings. In the first year, we observed a 30.8% reduction in IOP, followed by a relatively steady 28–30% reduction over time. Similarly, at three years, the mean number of medications was 0.6 ± 1.1, with a slight yearly increase reaching 1.4 ± 1.3 by 10 years. For phacocanaloplasty, these studies report IOP reductions of 15% to 43%, with mean IOP values between 13.6 and 15.6 mmHg and medication use ranging from 0.3 to 0.7. In our study, we observed a more moderate initial IOP reduction of 23.3%, which then stabilized around 30% at year 5. This smaller reduction may be attributed to the small patient cohort and the relatively low baseline IOP of 20.6 ± 5.9 mmHg. Medication use in our study was similar, at 0.4 ± 0.7 at three years, with a slow yearly increase up to 0.8 ± 1.1 at ten years. The greater IOP reduction and lower medication dependence in the later years may be attributed to the exclusion of patients who required secondary interventions.

Although some studies suggest that phacoemulsification alone would result in a long-term, mild IOP reduction of 1–2 mmHg [17,18], a meta-analysis from Jiang et al. found no statistically significant IOP reduction in canaloplasty vs. phacocanaloplasty, consistent with our findings [9]. In our study, the phacocanaloplasty group had, on average, about 0.7 mmHg lower IOP compared to the canaloplasty group; however, this difference was not statistically significant. Contrary to the theoretical expectation of lower early postoperative IOP in the phacocanaloplasty group due to the combined effect of cataract surgery, our data showed higher IOP at 1 month (16.5 ± 1.2 mmHg vs. 14.3 ± 0.7 mmHg for CP), possibly due to the small sample size or steroid response.

Our results demonstrate that canaloplasty performed using either the iTrack or Glaucolight microcatheters yields comparable IOP-lowering effects. Interestingly, phacocanaloplasty with Glaucolight demonstrated a greater hypotensive effect than with iTrack. We attribute this finding to the small sample size. Since Glaucolight is no longer commercially available, we routinely use a twisted 6/0 polypropylene suture, Onalene^®^ (Geuder, Heidelberg, Germany), for probing the SC when possible, as it is more cost-effective and previous studies have demonstrated comparable canulation rates to iTrack [19,20,21].

During combined procedures, phacoemulsification can be performed either through a shared incision or a separate temporal clear corneal incision. If a separate incision is preferred, phacoemulsification is performed first, followed by canaloplasty [22]. However, we favor the shared incision technique, as we consider it less traumatic to the eye.

Regarding safety, the incidence of intraoperative complications in our study was relatively low, which is consistent with the literature. Descemet perforation was the most common intraoperative complication, similar to previous reports [6,7,23]. Pullig et al. compared three groups who underwent canaloplasty with intact DW (group 1), canaloplasty with accidental rupture of the DW (group 2), and canaloplasty with a scheduled puncture of DW postoperatively (group 3) and found no statistically significant difference in the IOP reduction profile but bleb formation was significantly higher in groups 2 and 3 [24]. As canaloplasty is traditionally a blebless procedure, bleb formation is considered a postoperative complication. Bleb formation was reported up to 10% in the literature [11,25], and could be attributed to pathological changes in the sclera due to longstanding topical medication use, high myopia, or the inability to close the scleral flap in a watertight fashion [26]. These blebs are usually small and often have no effect on postoperative IOP values. To date, no bleb-related complications have been reported after canaloplasty [13,26]. While the impact of bleb formation on intraocular pressure (IOP) remains uncertain, some authors suggest that it might serve as a negative prognostic factor [27]. In our study, HDMD occurred in three cases (3.53%) while using the iTrack microcatheter, consistent with previous reports [23,27,28]. DMD is more commonly observed in the inferior quadrants, likely due to increased viscoelastic accumulation and/or uneven OVD distribution. This may weaken Descemet’s membrane at its termination at Schwalbe’s line [28], leading to detachment and possible blood reflux into the corneal stroma [27,29,30]. To mitigate this risk, we now routinely limit viscodilation to the ostia rather than performing a full 360° dilation. In our cases, HDMD developed in the inferior quadrants without affecting the visual axis and resolved spontaneously within 3–6 months without any further complications.

Up to 20% of patients undergoing canaloplasty require a laser goniopuncture due to IOP elevation in the first early postoperative months [16,20]. Interestingly, patients undergoing a combined procedure rarely need a postoperative laser treatment [30], which may be due to de deepening of the anterior chamber during and after surgery.

It is generally known that patients with PEXG are harder to manage with topical therapy, but there is little evidence to support a higher likelihood of re-operation and IOP decompensation after canaloplasty. Our results suggest that patients with PEXG are approximately five times more likely to require reoperation compared to those with POAG. However, this finding may not be as robust due to the small sample size, which limits the statistical power of the analysis. Brusini et al. followed patients with PEXG after canaloplasty in a retrospective case series. During the follow-up period, 61.2% of the patients had an abrupt IOP decompensation, most frequently observed 2 to 4 years after surgery, and 35.8% of the patients needed a second intervention [13]. The poorer prognosis of PEXG may be linked to its clinical characteristics, particularly its association with higher baseline IOP levels, more pronounced diurnal IOP fluctuations, and significant IOP spikes [31,32]. PXM obstructs gaps in the trabecular meshwork, facilitating the accumulation of pigment and debris [33], which in turn leads to blockage of the channels responsible for aqueous outflow into Schlemm’s canal. This phenomenon continues to progress even after successful surgery, compromising trabeculo-canalicular outflow over time. If canaloplasty is considered for the management of PEXG, a modified technique with suprachoroidal drainage may offer additional benefits by enhancing the uveoscleral outflow and further reducing the IOP [34]. Seuthe et al. demonstrated a significant reduction in IOP by 45.8% after 12 months and 45.1% after four years with a decrease in medications from 3.4 at baseline to 0.6 after 12 months and to 1.0 after four years [35].

In conclusion, our study demonstrates that canaloplasty and phacocanaloplasty are effective long-term surgical options for reducing IOP and medication need in OAG patients. Both procedures achieved sustained IOP reductions of approximately 30% over a 10-year follow-up, with comparable outcomes between surgical methods and low complication rates. While medication use gradually increased over time, it remained significantly lower than baseline levels.

The limitations and potential biases of this study include the lack of randomization, a small sample size, and its retrospective nature. The lack of significant differences between groups may also be influenced by the unequal sample sizes, which likely reduced statistical power to detect subtle differences. The 10-year duration represents a considerable timeframe during which some patients are lost to follow-up or mortality before study completion, thereby impacting data integrity and completeness.

## Figures and Tables

**Table 1:** Baseline data.

Variable	Descriptive Summary
Total eyes	85
Age (years)	
Mean ± SD	68.8 ± 8.1
Range	55–84
Sex: count (%)	
Female	49 (54.5)
Male	20 (45.5)
Eye treated: count (%)	
Right eye	43 (50.6)
Left eye	42 (49.4)
Combined cataract procedure: count (%)	
Yes (phacocanaloplasty)	20 (22.4)
No (canaloplasty)	65 (77.6)
Preoperative lens status	
Pseudophakic	31 (36.5)
Phakic	54 (63.5)

**Table 2:** Adjusted IOP and medication use after canaloplasty (CP) vs. phacocanaloplasty (CP+Phaco). Values represent means ± standard errors from a linear mixed model, adjusted for time, lens status, and exclusion of cases requiring reoperation (see Supplementary Table S1 for raw IOP data and full unadjusted metrics). Medication use is reported as raw means due to clinical relevance and absence of significant covariates in the GLMM.

Follow Up Time	Adjusted Mean IOP ± SE (CP Group)	Mean Meds ± SD CP Group	N	Adjusted Mean IOP ± SE (CP+Phaco)	Mean Meds ± SD CP+Phaco Group	N
Preoperative	22.1 ± 0.9	2.4 ± 1.0	65	20.4 ± 1.5	2.4 ± 1.0	20
1 month	14.3 ± 0.7	0.0	65	16.5 ± 1.2	0.0	20
3 months	14.2 ± 0.5	0.0	65	14.6 ± 0.9	0.0	20
6 months	14.2 ± 0.4	0.0 ± 0.3	65	13.9 ± 0.7	0.0 ± 0.6	20
9 months	14.3 ± 0.5	0.0 ± 0.4	65	15.1 ± 0.9	0.2 ± 0.6	20
1 year	15.3 ± 0.5	0.1 ± 0.5	65	15.6 ± 1.0	0.3 ± 0.9	20
2 years	15.6 ± 0.5	0.5 ± 0.9	62	15.5 ± 0.8	0.3 ± 0.6	20
3 years	15.3 ± 0.4	0.6 ± 1.1	59	15.1 ± 0.7	0.4 ± 0.7	19
4 years	15.8 ± 0.4	0.7 ± 1.1	56	14.8 ± 0.7	0.9 ± 1.4	19
5 years	15.7 ± 0.5	0.8 ± 1.1	55	14.3 ± 0.8	0.9 ± 1.4	19
6 years	15.9 ± 0.5	1 ± 1.2	54	15.1 ± 0.9	0.8 ± 1.1	17
7 years	16.0 ± 0.6	1.1 ± 1.2	54	14.3 ± 1.0	0.8 ± 1.1	17
8 years	16.0 ± 0.6	1.3 ± 1.3	54	13.7 ± 1.0	0.8 ± 1.1	17
9 years	15.8 ± 0.6	1.3 ± 1.2	54	13.6 ± 1.0	0.8 ± 1.1	17
10 years	15.9 ± 0.7	1.4 ± 1.3	52	14.2 ± 1.2	0.8 ± 1.1	17

**Table 3:** Surgical success rates for group 1 and group 2.

Success Rates at 1, 3, 5, 7, and 10 Years After Surgery for Groups 1 and 2									
Complete success (IOP ≤ 18 mm Hg without medication) at the given follow-up time point; number of eyes that fulfilled success criteria/n (%)									
Group 1 (Canaloplasty)					Group 2 (Phacocanaloplasty)				
Year 1	Year 3	Year 5	Year 7	Year 10	Year 1	Year 3	Year 5	Year 7	Year 10
89.2% (58/65)	53.9% (35/65)	49.2% (32/65)	38.5% (25/65)	29.2% (19/65)	85.0% (17/20)	60.0% (12/20)	50.0% (10/20)	45.0% (9/20)	45.0% (9/20)
Qualified success (IOP ≤ 18 mm Hg with or without medication) at the given follow-up time point; number of eyes that fulfilled success criteria/n (%)									
Year 1	Year 3	Year 5	Year 7	Year 10	Year 1	Year 3	Year 5	Year 7	Year 10
95.4% (62/65)	83.1% (54/65)	80.0% (52/65)	75.4% (49/65)	66.2% (43/65)	90.0% (18/20)	80.0% (16/20)	75.0% (15/20)	70.0% (14/20)	70.0% (14/20)

## Data Availability

The datasets generated during and analyzed during the current study are available in the figshare repository, DOI: 10.6084/m9.figshare.28518254.

## Supplementary Materials

The following are available online at https://www.mdpi.com/article/10.3390/jcm14072481/s1, Table S1: Raw IOP Data and Full Unadjusted Metrics.
