# Supplementary material for: Ten-Year Results After Canaloplasty and Phacocanaloplasty
Source: J Clin Med. 2025 Apr 4;14(7):2481. doi: 10.3390/jcm14072481 (PMC11989742; doi:10.3390/jcm14072481)
Supplement: Supplementary file 1 [file jcm-14-02481-s001.zip › jcm-3533492-supplementary.pdf]

Supplementary Table S1. Raw IOP Data and Full Unadjusted Metrics

Follow Up Time	Mean IOP±SD CP Group	Median	Mean Meds±SD CP Group	N	Mean IOP±SD CP+phaco	Median	Mean Meds±SD CP+phaco Group	N
Preoperative	22.1±6.1	21.0	2.4±1	65	20.6±5.9	19.0	2.4±1	20
1 month	14.3±4.5	14.0	0	65	16.4±6.1	14.5	0	20
3 months	14.2±3.3	14.0	0	65	14.8±5.9	12.5	0	20
6 months	14.2±3.5	14.0	0±0.25	65	14.0±3.6	13.0	0±0.6	20
9 months	14.3±3.3	14.0	0±0.37	65	15.2±6.5	13.5	0.2±0.62	20
1 year	15.3±3.8	14.0	0.1±0.5	65	15.8±6.0	15.0	0.3±0.9	20
2 years	15.5±2.9	15.0	0.5±0.9	62	15.6±5.6	14.5	0.3±0.6	20
3 years	15.3±2.8	15.0	0.6±1.1	59	15.1±4.0	15.0	0.4±0.7	19
4 years	15.7±3.2	15.5	0.7±1.1	56	14.7±3.7	14.0	0.9±1.4	19
5 years	15.6±3.6	16.0	0.8±1.1	55	14.5±3.3	14.0	0.9±1.4	19
6 years	15.8±3.6	16.0	1±1.2	54	14.8±4.4	14.0	0.8±1.1	17
7 years	16.1±4.1	15.0	1.1±1.2	54	13.7±3.5	14.0	0.8±1.1	17
8 years	16.0±4.4	15.0	1.3±1.3	54	13.0±3.5	13.0	0.8±1.1	17
9 years	15.8±5.2	15.0	1.3±1.2	54	13.5±2.9	14.0	0.8±1.1	17
10 years	15.9±6.5	15.0	1.4±1.3	52	14.1±3.1	14.0	0.8±1.1	17
